# Advancements and mechanisms of stem cell-based therapies for spinal cord injury in animals

**DOI:** 10.1097/JS9.0000000000001074

**Published:** 2024-01-24

**Authors:** Bhabesh Mili, Om Prakash Choudhary

**Affiliations:** a Department of Veterinary Physiology and Biochemistry, College of Veterinary Sciences and Animal Husbandry, Central Agricultural University (I), Jalukie, Peren, Nagaland; b Department of Veterinary Anatomy, College of Veterinary Science, Guru Angad Dev Veterinary and Animal Sciences University (GADVASU), Rampura Phul, Bathinda, Punjab, India

**Keywords:** central nervous system (CNS), mesenchymal stem cells (MSCs), neurotrophic factors, spinal cord injury (SCI), stem cells

## Abstract

Spinal cord injury (SCI) is a neurodegenerative disorder of the central nervous system that can lead to permanent loss of sensation and voluntary movement beyond the affected area. Extensive preclinical and clinical trials have been conducted to evaluate the safety and effectiveness of stem cells for the treatment of various central nervous system diseases or disorders, including SCI. However, several challenges hinder nerve cell regeneration in the injured spinal cord, such as extensive cell loss, limited neural cell regeneration capacity, axonal disruption, and the presence of growth-inhibiting molecules, particularly astroglial scarring or glial scars at the injury site in chronic cases. These obstacles pose significant challenges for physicians in restoring normal motor and sensory nerve function in both humans and animals following SCI. This review focuses on SCI pathogenesis, the mechanisms underlying the therapeutic potential of mesenchymal stem cells in SCI, and the potential of stem cell-based therapies as promising avenues for treatment. This review article also included relevant preclinical and clinical data from animal studies.

## Spinal cord injury

Highlights

- Neuropathology of spinal cord injury (SCI) at the cellular level.
- Different types of stem cells and the advantages of mesenchymal stem cells (MSCs) over other stem cell types in cell-based therapies.
- Therapeutic efficacy of MSCs harvested from different sources in treating SCI in animals.
- Challenges and future optimization approaches for the effective clinical practice of MSCs for treating SCI in animals.

Spinal cord injury (SCI) is a leading cause of disrupted communication between the CNS and the rest of the body, resulting in a significant risk of physical disability. This condition affects humans and animals, with common causes including traffic accidents, falls from heights, sports and recreational activities, violence, and work-related accidents^1^. Cervical vertebral compressive myelopathy (CVCM or Wobbler syndrome) in horses^2^ and intervertebral disc disease (IVDD) in dogs^3^ are the prevalent causes of spinal ataxia in animals. IVDD is a neurological syndrome characterized by SCI due to disc herniation^3^.

The neuropathology of SCI unfolds gradually after mechanical trauma or following injury and is characterized by two phases: the initial mechanical injury (primary injury) and subsequent damage to surrounding tissues (secondary injury). SCI is categorized as acute (lasting 1–2 days), sub-acute (lasting 1 day to 8 weeks), or chronic (lasting months to years), depending on the time frame^4^. Physical trauma to the spinal cord disrupts the blood-brain barrier, causes vascular injury and rapid cell death, and triggers an inflammatory response at the site of injury. Inflammatory cells, such as neutrophils, macrophages, lymphocytes, and microglia, migrate to the lesion and release inflammatory cytokines, exacerbating spinal cord neuropathology by demyelinating nerve fibers and axons^5–7^. At the cellular level, oxidative stress leads to DNA damage, lipid peroxidation, protein oxidation, and mitochondrial dysfunction in both neurons and oligodendrocytes^6–8^, ultimately causing progressive demyelination of nerve fibers and exposed axons^9^.

In the sub-acute phase of SCI, pathogenic processes such as inflammation, ischemia, hypoxia, and anoxia persist over days to weeks. These processes lead to lipid peroxidation, cell death induced by free radicals, disruption of ion channels, axonal demyelination, necrosis, and apoptosis, further impairing nerve tissue function^8^. In chronic cases, or when SCI lasts for months or years, more macrophages and astrocytes migrate to the injury site, forming a glial scar. This scar acts as a long-term indicator of SCI and separates the injured spinal cord from healthy adjacent tissues, inhibiting axonal sprouting and regeneration^10,11^. Studies also indicate that extracellular matrices, combined with debris from dying neurons, inflammatory substances, and toxic metabolites from dead cells, contribute to secondary tissue damage around the nerve tissue. These factors hinder cell migration and axonal regeneration. Therefore, to restore normalcy after SCI, it is essential to focus on preventing secondary tissue injury based on neuropathology. Subsequently, repairing damaged axons and synaptic connections and developing a functional neural network circuit have become key priorities^12^.

## Stem cells

Stem cells are unspecialized cells in the mammalian body. They can renew themselves, differentiate into specific cell types with specific functions, and exhibit clonogenic potential both *in vivo* and *in vitro* ^13^. Stem cells renew themselves through genetic pathways influenced by signals from their microenvironment called niches. These properties offer hope for the treatment of incurable diseases via transplantation. In 1991, Caplan introduced stem cell therapy, which has gained significant attention and is now used in hospitals to treat certain diseases in humans and animals. Stem therapy involves controlled stem cell culture and laboratory derivations. Different types of stem cells are broadly categorized into embryonic, fetal, and adult stem cells (ASCs). They can also be classified based on their origin in embryos, fetuses, and adults^14^. Additionally, stem cells are categorized according to their differentiation potential, such as totipotency, pluripotency, multipotency, or unipotency. Embryonic stem cells (ESCs) are derived from the inner cell mass (ICM) of the blastocyst, possess the highest developmental potential, and can become all cell types in the body, making them pluripotent. ASCs are found in adult tissues. These cells have been found to have broad plasticity that allows them to differentiate across tissue lineage boundaries to give rise to cell types of other lineages. ASCs have been identified in various locations, including the spinal cord, fat tissue, bone marrow, placentas, brain, olfactory tissue, umbilical cord blood, and connective tissue in various organs^15^. Fetal stem cells are primitive cell types in fetal organs. These cells can be collected after birth and exhibit robust division potential and pluripotency^14^. Some scientists believe that adult and fetal stem cells may originate from ESCs. They suggested that the few stem cells in adult organs could be leftover ESCs that did not fully become organs and could now help with tissue repair when injuries occur.^16^. Thus, these cells act as repair systems because they can divide indefinitely to replace other cells.

## Totipotent stem cells

Totipotency refers to the remarkable ability of a single cell to divide and differentiate into an entire organism. In mammals, particularly placental animals, totipotent stem cells possess the extraordinary ability to transform into any cell type within an adult body, including those that make up the extra-embryonic membranes, enabling the development of a complete organism. In nature, only the zygote and blastomere at the very early cleavage stage exhibit totipotency^17^. Notably, cells originating from fertilized eggs retain the potential to give rise to all embryonic and extra-embryonic cells, although their full developmental capacity in vitro remains uncertain^18^.

## Pluripotent stem cells (PSCs)

PSCs have a remarkable ability to differentiate into the three primary tissue types (endoderm, ectoderm, and mesoderm), but cannot develop into cells with extra-embryonic membranes, unlike ESCs. They are naturally present for a brief period and include later-stage blastomeres, the ICM of the blastocyst, and the ICM-derived epiblast. When placed under the right culture conditions, ICM cells can give rise to pluripotent ESCs in vitro. PSCs are categorized into various groups based on their origin, including ESCs and induced pluripotent stem cells (iPSCs) (derived from somatic cells). iPSCs are produced by reprogramming somatic cells to become virtually identical to ESCs. This breakthrough was achieved by expressing specific reprogramming factors (OCT4, KLF4, SOX2, and c-MYC) in mouse fibroblasts, resulting in the generation of iPSCs^19^.

## Multipotent stem cells

Multipotent stem cells, such as mesenchymal stem cells (MSCs), are found in various body organs. These stem cells, descendants of pluripotent cells, serve as precursors to specialized cell lines, such as blood cells, skin cells, and neural cells^15^. Multipotent stem cells are partially undifferentiated and are present in nearly all adult tissues. They possess the capacity to generate a limited range of specialized cell types, typically those closely related to a particular family of cells. These stem cells are less versatile and specialized. For instance, MSCs are primarily derived from the bone marrow^20^. They can differentiate into mesodermal lineage cells, including osteoblasts, adipocytes, and myoblasts^21–24^, as well as neurons or neuron-like cells^25,26^.

## Unipotent stem cells

Unipotent stem cells are specialized cells capable of producing only one specific cell type yet retaining the ability to self-renew. Examples of unipotent stem cells include limbic stem cells^27^, testicular spermatogonial stem cells^28^, and neural stem cells (NSCs)^29,30^. Spermatogonial stem cells play a crucial role in the initiation of sperm production^28^. Similarly, NSCs are versatile undifferentiated cells within the CNS. They possess the capacity for self-renewal and can generate various specialized CNS cells, including neurons, astrocytes, and oligodendrocytes^29,30^.

## MSCs: cell-based therapy advantages

Cell-based therapies utilizing stem cells have been explored to treat various human and animal diseases and injuries^31^. However, not all stem cell types are equally suitable, owing to certain limitations. For instance, iPSCs can have genetic and epigenetic defects with teratogenic risks^32^, while NSCs have limited sources^33^, and ESCs raise ethical concerns because they require embryos.

MSCs are the preferred choice of cell-based therapies in regenerative medicine. Their advantages include the following:

- Versatility in sources: MSCs can be obtained from various tissue sources without ethical concerns, including bone marrow, fat, muscle, dental pulp, brain, umbilical cord blood, adipose tissue, and amniotic fluid, in both humans and animals^34–36^. These cells are easy to isolate and expand *in vitro* in order to obtain a sufficient amount for clinical application and can also be preserved *in vitro*. These cells can maintain their stem potential after cryopreservation^37^. Furthermore, these cells have a potential autologous origin compared with other types of stem cells, allowing recipients to avoid alloantigen responses.
- Differentiation potential: MSCs can differentiate into multiple cell lineages like osteoblasts, chondrocytes, adipocytes, fibroblasts, neurons, glial cells, etc., similar to ESCs, and possess a high proliferation rate, making them appealing for therapeutic applications^38,39^.
- Distinct immunomodulatory effects: The most attractive aspect of MSCs in cell-based regenerative medicine is their powerful immunomodulatory properties, which allow them to transplant MSCs to escape immune system recognition and modulate host defense mechanisms^40^. Immunosuppression has been suggested to occur through cell-cell contact and secreted paracrine factors via the expression of adhesion molecules such as vascular cell adhesion molecule 1 (VCAM1), and the secretion of proinflammatory cytokines such as interferon-gamma (IFN-γ), and tumor necrosis factor-alpha (TNF-α), interleukin-6 (IL-6), and prostaglandin E2 (PGE2)^41–44^. Furthermore, MSCs exhibit low MHC class I antigen expression and the absence of MHC class II, resulting in paracrine components with immunomodulatory properties^45–48^. Additionally, MSCs suppress allogeneic T cell proliferation and B lymphocyte attenuation^49,50^.
- Homing capabilities: MSCs possess remarkable homing ability, which refers to their capacity to migrate and target specific tissues or injured areas within the body^51^. This homing ability of MSCs after transplantation is typically associated with cytokine outbursts^52,53^. This innate ability enhances the therapeutic potential of stem cells for treating various conditions, including injuries and degenerative diseases.
- Paracrine activity: MSCs, in addition to their ability to transform into various cell types, display other valuable functions for clinical applications. Beyond their role as progenitors, MSCs possess nonprogenitor functions, demonstrating their capacity to impact resident cells and tissues^54^. This effect is exerted through the secretion of paracrine and neurotrophic factors by MSCs. MSCs secrete paracrine and neurotrophic substances with anti-inflammatory, antiapoptotic, antioxidative, angiogenic, axonal development, and neuroregenerative properties^55–58^, play crucial roles in modulating cellular activities and tissue functions, making MSCs promising candidates for a wide range of therapeutic interventions.

## Underlying therapeutic mechanisms of MSCs in SCI

**Figure 1:**
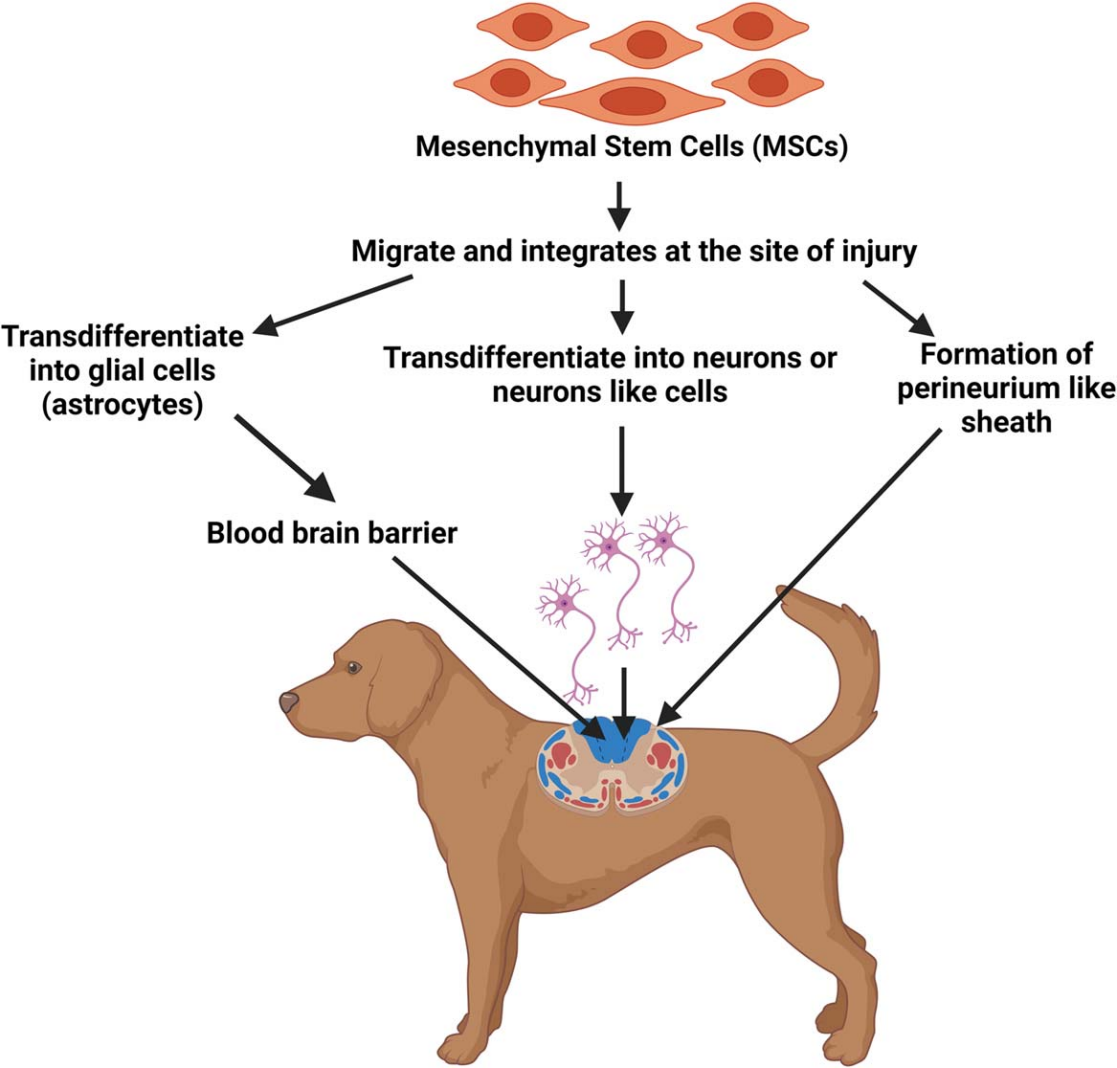
Mesenchymal stem cells directly transdifferentiate into nerve and glial cells to promote nerve regeneration in spinal cord injury.

**Figure 2:**
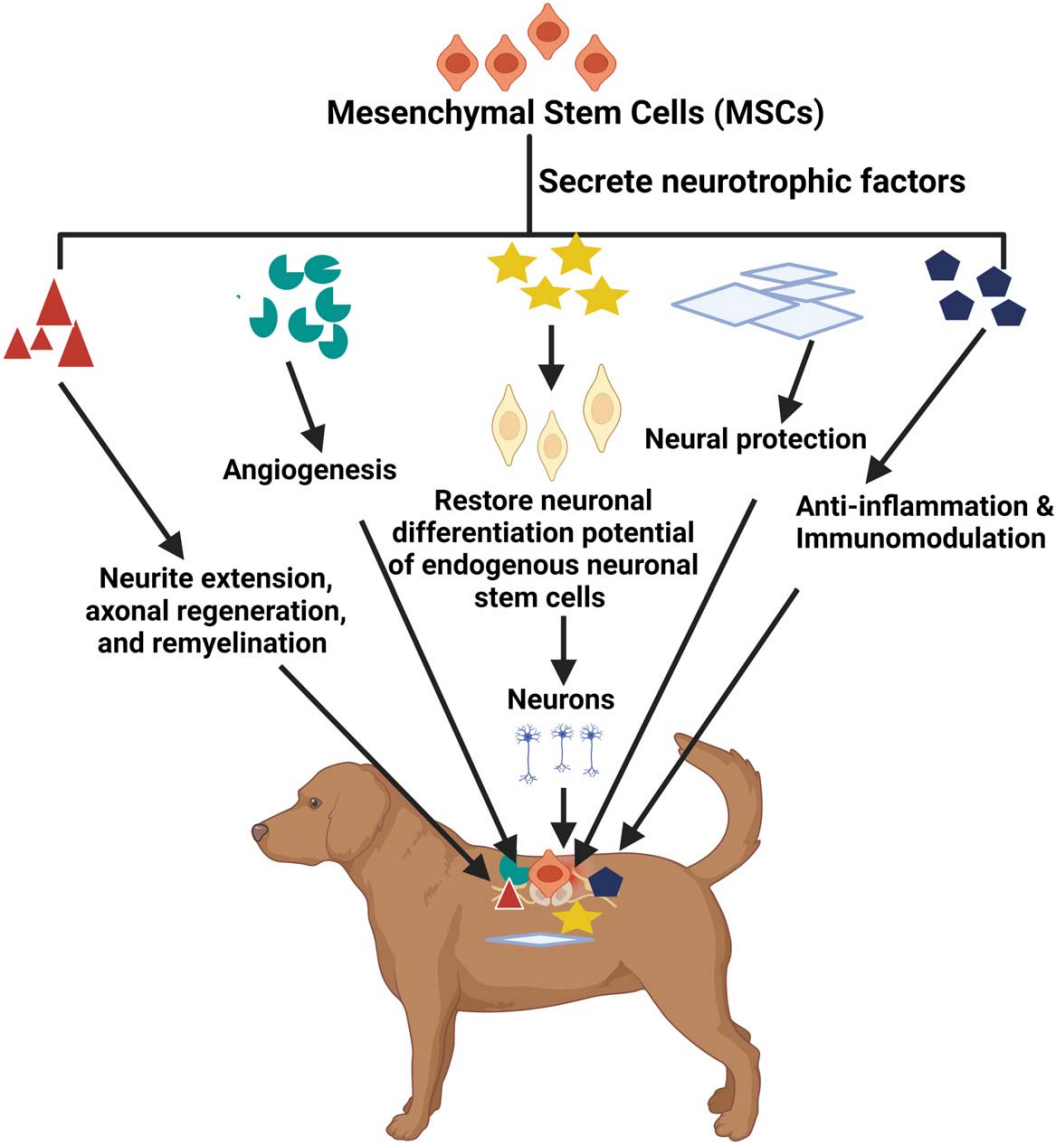
Mesenchymal stem cells promote nonprogenitor function in nerve regeneration in spinal cord injury.

Transplanted stem cells, particularly MSCs, have complex mechanisms for restoring normalcy after SCI. These mechanisms can be classified as direct (Fig. 1) and indirect by enhancing nonprogenitor function (Fig. 2) at the site of injury^12^. Direct effects involve the recruitment of new neurons and glial cells to the injury site, potentially aiding neuronal regeneration^59–61^. MSCs have shown the ability to transdifferentiate into nervous system cells, further supporting this regeneration process^60–64^. However, in injured spinal cords, most NF160- and NeuN-positive neurons are derived from the spinal cord’s own neural progenitor cells^61^. *In vitro* studies have shown that MSCs express neuronal markers, indicating their potential for neural and glial differentiations. MSCs at the injury site can also create a protective sheath, shielding regenerating nerve fibers from oxidative damage, and providing support for native brain cells^63^. Additionally, these transplanted cells help to reduce glial scars at the injury site, creating a favorable microenvironment for brain regeneration. Furthermore, these transplanted cells help reduce glial scars at the injury site, promoting the microenvironment necessary for brain regeneration^65–67^.

Transplanted MSCs play a vital role in activating endogenous NSCs and in restoring their neurological capabilities. Both undifferentiated transplanted MSCs and certain secreted neurotrophic factors, including glial cell line-derived neurotrophic factor (GDNF), nerve growth factor (NGF), brain-derived neurotrophic factor (BDNF), ciliary neurotrophic factor, and Basic Fibroblast Growth Factor (bFGF), are essential for reinvigorating the differentiation potential of endogenous NSCs^68^. Consequently, the loss of neurons cannot be replenished by NSCs of the nervous system’s own^69^. Moreover, when activated, endogenous NSCs at the injury site are more likely to differentiate into astrocytes^70^, thus playing a vital role in restoring neuronal regeneration following SCI.

In contrast to direct effects, many authors have reported that MSCs therapy primarily results in indirect environmental modification rather than the direct transformation of migrated MSCs into functional neurons or glial cells^71,72^. MSCs play a crucial role in SCI repair by secreting the antiapoptotic protein B-cell lymphoma 2 (Bcl-2) while preventing the release of proapoptotic proteins such as Bcl-2-associated X protein and caspase 3^73,74^. They also release neurotrophic substances, including BDNF, bFGF, NGF, TGF-β, VEGF, GDNF, and SDF-1, which are significantly upregulated in MSC-transplanted animals compared to normal or control groups^60^. Among these, NGF and NT-3 play key roles in neural differentiation and proliferation of neural progenitor cells^72^, whereas SDF-1 supports the survival of neurons and aids those damaged by injury or inflammation^75–77^. Furthermore, MSCs release neurotrophic factors such as monocyte chemoattractant protein-1 and granulocyte-macrophage colony-stimulating factor, which protect neurons and promote the recruitment of monocytes to clear myelin debris, facilitating neurite extension, axonal regeneration, and remyelination^78^. Myelin regeneration, restoring protective myelin sheaths around damaged axons, is a targeted mechanism for enhancing recovery from SCI^79^. MSCs also produce various neurotrophic factors, including BDNF, VEGF, NGF, GDNF, bFGF, and EGF, which support nervous tissue regeneration by promoting synapse formation and myelination. These combined mechanisms underscore the potential therapeutic benefits of MSC transplantation in SCI treatment^12^.

Transplanted MSCs play a crucial role in promoting tissue repair by indirectly reducing the inflammatory responses at the injury site. Inflammation can have beneficial and harmful effects on the healing process^80–82^, making MSC transplantation valuable for immunomodulation. This can involve dampening detrimental inflammation or promoting beneficial aspects of the inflammatory response^79^. While it is often challenging to determine whether transplanted cells directly alter inflammation due to potential neuroprotective mechanisms^83^, some studies suggest that MSCs achieve immunomodulation through the secretion of anti-inflammatory cytokines and inhibition of proinflammatory cytokines, such as TNF, IFN-gamma, and IL-6. These interactions between transplanted and immune cells are critical for optimizing the healing process after SCI^8,65,66,84^.

Spinal cord injuries often lead to widespread blood vessel loss, creating local hypoxia in the injury area^79,85^. Transplanted MSCs release molecules that protect the existing blood vessels and promote the formation of new molecules via vascular angiogenesis. This process involves the secretion of factors such as TIMP metallopeptidase inhibitor-1, VEGF, HGF, PDGF, IL-6, and IL-8. VEGF plays a critical role in vascular growth and development as well as in neurotrophic and neuroprotective functions, independent of vascular effects^77,86^. Ultimately, these factors increase blood vessel density at the SCI site, improving local blood supply and potentially enhancing the healing process^8,87^.

## Current status of stem cell therapeutics for treating SCI in animals

Various types of stem cells like umbilical cord blood-derived MSCs^59^, BM-MSCs^60,88–91^, neural-induced-MSCs^72^, fetal bone marrow-derived stem cells^92^, human NSCs^93^, amniotic membrane-derived stem cells^94^, canine exfoliated deciduous teeth-derived stem cells^95^, human immature dental pulp stem cells^96^, neural progenitor cells from canine-iPSCs^97^, human MSCs^98^, and human umbilical cord-derived MSCs^99,100^, have been explored in clinical and preclinical investigations for SCI treatment in both animals. Among these, BM-MSCs^60,63,88–91,101–103^ and adipose-derived MSCs (AD-MSCs)^84,104–110^ have been extensively used in canine SCI studies. The effectiveness of stem cell therapy for SCI in animals is summarized in Tables 1 and 2. The outcomes of stem cell therapy for SCI in animals vary significantly, likely because of the complex and unpredictable nature of SCI pathophysiology, differences in stem cell types, their source, and mode of administration, among other factors.

**Table 1:** Preclinical studies of MSCs derived from different sources in the treatments of SCI in animals.

S. No.	Type of injury	Types of animals and age	Treatment group	Control group	Cell transplantation methods	Types of stem cells	Number of stem cells	Therapeutic efficacy	References
1	Experimentally induced SCI.	Animal: Adult Mongre dog	Three groups with *n*=5 each (G1-rmhGCSF, G2-UCBMSC: stem cells and G3-UCBG: both stem cells and rmhGCSF)	Two groups with *n*=5 each (CN: No treatment and CP: Injected media)	Intralesional transplantation of stem cells. Subcutaneous injection of recombinant methionyl human granulocyte colony-stimulating factor (rmhGCSF)	Canine umbilical cord blood-derived mesenchymal stem cells (UCB-MSCs)	1×10^6^	New neuronal formation in injured spinal cord structures in the UCB-MSC and UCBG groups. Somatosensory evoked potentials revealed a significant increase in nerve conduction velocity in both groups. Moreover, both groups showed improved pelvic limb weight-bearing ability, which increased from 10 to 50% after 8 weeks.	^59^
2	Experimentally induced acute SCI	Animal: Beagle dog Age: 1–4 years	Two groups with *n*=10 each (Autologous and allogeneic)	Treated with PBS (*n*=2).	Intralesional transplantation.	Autologous and allogeneic bone marrow-derived mesenchymal stem cell (BM-MSC)	1×10^7^	Both allogenic and autologous groups showed significantly higher mRNA expression of neurotrophic factors than the control group. Notably, autologous MSC transplantation resulted in greater functional recovery after SCI than allogeneic BM-MSCs transplantation. Within five weeks, dogs receiving autologous BM-MSCs could stand and lift their trunk, whereas those treated with allogeneic BM-MSCs exhibited only a sensory response	^60^
3	Experimentally induced acute SCI	Animal: adult beagle dog	Four groups with *n*=4 each (*n*=16)	No treatments (*n*=4)	Intralesional transplantation.	BM-MSCs, AD-MSCs, and WJ-MSCs), and UCB-MSCs	6×10^6^	Transplanted MSCs survive, migrate toward neural cells at the injury site, and play a crucial role in preserving axons and neural cells, resulting in improved hind limb function after SCI. Significant functional enhancements were evident in the treatment groups after 8 weeks. UCSCs showed superior nerve regeneration, neuroprotection, and reduced inflammation compared with other MSCs	^61^
4	Experimentally induced acute SCI	Animal: adult beagle dog	Three groups with *n*=2 each) (Evaluated G- 2 weeks after transplantation, G-4, 4 weeks after transplantation, and G-5 4 weeks after multispot transplantations	Two groups with *n*=2 each (Evaluated G-1 2 weeks and G-3 4 weeks after sham transplantation)	Percutaneous transplantation	Human UCB-MSCs	10^6^	In G-4 and G-5, three out of four dogs showed gradual improvement in hind limb movement starting 3 weeks after receiving cellular transplants. The transplanted UCB-derived MSCs tested positive for neuronal markers (NeuN, glial fibrillary acidic protein, and von Willebrand factor), suggesting their potential contribution to neural repair	^99^
5	Acute SCI	Animal: Adult dog	*n*=5	*n*=3 Treated with only PBS	Intralesional transplantation.	Allogenic adipose-derived stem cells (ASC).	1×10^6^	In the stem cell group, nerve conduction velocity (based on SEP) was significantly improved compared to that in the control group. Immunohistochemical analysis revealed the presence of GFAP, Tuj-1, and NF160 in cells derived from implanted ASCs. These findings suggest that the enhanced neurological function following ASC transplantation in dogs with spinal cord injury may be attributed, at least in part, to neural differentiation of the implanted stem cells	^71^
6	Experimentally induced SCI.	Animal: Beagle dogs Age: 2–3 years.	Two treatment groups with *n*=4 each ( one group treated with stem cells and the other one with both stem cells and chABC)	Two l groups with *n*=4 each (one group treated with PBS only and the other with chondroitinase ABC (chABC)	Intralesional transplantation.	Canine Ad-MSCs	1×10^7^	Dogs treated with Ad-MSCs, either alone or with chABC, showed significantly improved functional recovery eight weeks after transplantation compared to the control and chABC-only groups. The combination of Ad-MSCs and chABC enhanced the expression of markers associated with nerve regeneration, including digested CSPGs (2B6), β3-tubulin, and NF-M. However, the levels of COX2 and tumor necrosis factor were higher in the treatment groups than in the control group	^108^
7	Experimentally induced SCI.	Animal: Beagle dog Age: 2–3 years	Two groups with *n*=4 each (AD-MSCs, and AD-MSCs + MPSS)	Two groups with *n*=4 each (control and MPSS)	Intravenous injection	Allogenic AD-MSCs	1×10^7^	Early intravenous injection of AD-MSCs after acute SCI may prevent additional cellular damage by enhancing antioxidative and anti-inflammatory processes with no observed side effects. Both the AD-MSCs and AD-MSCs + MPSS groups showed improved hind limb movements after seven days. Hematoxylin and eosin staining revealed fewer hemorrhages and less microglial infiltration in the AD-MSCs group. All treatment groups demonstrated reduced levels of 4-hydroxynonenal, cyclooxygenase-2, interleukin-6, tumor necrosis factor-alpha, and phosphorylated-signal transducer and activator transcription 3.	^84^
8	Experimentally induced SCI	Animal: Beagle dogs Age: 6–8	Two groups (one received MSC-derived neural network tissue and survived for 6.5 months (*n*=6) and the other group received MSC-derived neural network tissue and survived for two months (*n*=3)	Two groups (one group without scaffold (*n*-3) and the other group received gelatin sponge scaffold)	Intralesional implantation	MSC-derived neural network scaffolds	—	Transplanting neural network tissue derived from BM-MSCs into dogs with complete SCI holds the potential for establishing a functional ‘neuronal relay’, aiding in the recovery of motor function in paralyzed limbs. BM-MSCs overexpressing Schwann cells demonstrated notable effectiveness in fully restoring neural circuits in paralyzed limbs, offering a promising approach for treating SCI and promoting neural recovery in affected animals	^63^ Wu *et al.*, 2018
9	Experimentally induced SCI	Animal: Beagle dog age: 1–2 years	Three groups with *n*=4 each (AD-MSCs over expressed brain-derived neurotrophic factor (BDNF), AD- MSCs over expressed heme oxygenase-1 (HO-1) and the other group combination of both)	Treated (*n*=4) with PBS	Intralesional transplantation.	AD-MSCs	1×10^7^	Dogs treated with a combination of BDNF-overexpressing AD-MSCs and heme oxygenase-1 showed significantly improved hind limb function compared to those treated with AD-MSCs alone. The combined therapy group exhibited notable neuroregeneration, as evidenced by increased expression of Tuj-1, NF-M, and GAP-43, along with reduced levels of inflammatory markers such as IL-6 and TNF-α. Additionally, there was an increase in the expression of interleukin-10 (IL-10)	^106^
10	SCI due to cervical ventral interbody fusion	Animal: Horse Age: 6 years	Two groups ( humanely killed (euthanized) at 48 hours (*n*=4) and the other group at 14 days (*n*=4)	—	Intramedullary transplantation	Allogeneic umbilical cord-derived mesenchymal stem cells (UC-MSC	10×10^6^	Intramedullary transplantation of UC-MSCs during CVIF surgery, guided by fluoroscopy, effectively reduces the risk of SCI and ataxia compared with endoscopy. UC-MSCs can persist in the spinal cord for up to 14 days, promoting angiogenesis and contributing to demyelination in local spinal tissue	^111^
11	Experimentally induced SCI	Animal: Beagle’s dog.	Treated (*n*=6) with Collagen/heparin sulfate scaffold (CHSS) with MSCs	No treatment (*n*=6)	Intralesional transplantation	Human mesenchymal stem cells (hMSCs)	1×10^7^	Dogs treated with CHSS and MSCs showed significantly better hind limb movement, standing, and walking abilities than the control group. This approach led to increased axonal regeneration, enhanced electrophysiological responsiveness, and improved locomotor recovery in dogs with acute complete spinal cord transection, indicating its potential as a promising treatment option	^98^

**Table 2:** Clinical application of MSCs derived from different sources in the treatments of SCI in animals.

S. No.	Type of injury	Number, types of animals and age	Number of treatment group	Number of control group	Cell transplantation methods	Types of stem cells	Number of stem cells	Therapeutic efficacy	References
1.	Chronic SCI	Animal: Boxer cross-bred dog Age: 6 months	*n*=1	—	Intralesional implantation with thermoreversible gelation polymer (TGP) followed by intravenous transfusion on the 19th postoperative day	Autologous bone marrow mononuclear cells (BM-MNCs)	First dose: 20 ×10^6^ Second dose: 4.16 × 10^6^	In a dog with chronic complete SCI, a single intrathecal injection followed by postoperative intravenous infusion of BM-MNCs showed improved functional recovery after spinal cord injury. By day 53, motor and sensory nerve activities and pelvic gait movements were observed. By day 180, the dog fully regained pelvic gait movements with no recurring neurological issues	^88^
2.	Accidental SCI	Animal: Cat Age: 1.5 years	*n*=1	—	Intralesional implantation with liquefied type I collagen	Autologous bone marrow-derived mesenchymal stem cells (BM-MSCs)	7×10^8^	Stem cell therapy followed by physiotherapy significantly improved the clinical condition of the cat with a spinal cord lesion. By the seventh day after transplantation, there was a gradual recovery in the panniculus reflex and both superficial and deep pain. However, persistent low proprioceptive reflexes and ataxic hind limb movements with hyperreflexia were observed	^112^
3.	Chronic SCI	Animal: Dog Age:2–4 years	*n*=4	—	Intralesional implantation	Autologous bone marrow-derived mesenchymal stem cells (BM-MSCs)	1×10^6^	All dogs exhibited progressive healing of the panniculus reflex and reduced superficial and deep pain response. Notably, three out of four animals demonstrated significant improvements in mobility at 18 months after transplantation	^89^
4.	Chronic SCI	Animal: Dog age: 2–10 years	*n*=7	—	Intramedullary transplantation	Allogenic fetal bone marrow-derived stem cells (BM-MSCs)	1×10^6^	All dogs with chronic SCI displayed improved locomotor and sensory functions 90 days after surgery. They demonstrated increased hind limb movement, the ability to stand upright, and the ability to take small steps. Additionally, seven dogs regained tail tone, five exhibited pain reflexes, and five showed a return of defecation capabilities	^92^
5.	Chronic SCI	Animal: Dog Age:2.5 months to 8 years	*n*=13	—	Percutaneous implantation	Autologous neurogenically-induced bone marrow-derived mesenchymal stem cells (NIBM-MSCs)	5.0×10^6^ second dose repeated after 21 days	Among the dogs studied, two showed improvements in gait, nociception, proprioception, somatosensory evoked potentials, and motor impulses, whereas six dogs exhibited only gait improvement. However, no improvement was observed in any of the five dogs	^101^
6.	Acute SCI (Acute thoracolumbar intervertebral disk disease)	Animal: Dog age: 5.32 ± 1.87 years	*n*=9	—	Intralesional transplantation	Allogenic Adipose-derived mesenchymal stem cells (AD-MSCs)	1×10^7^	Among the nine dogs, five fully recovered (56.6%), two regained deep pain perception with mild ataxia (22.2%), and two did not recover. MSC transplantation leads to improved clinical signs by modifying the inflammatory environment, enhancing endogenous nerve cell survival, reducing regulatory signaling molecules associated with glial scar formation, and facilitating partial differentiation into neural cells	^109^
7.	Chronic SCI	Animal: Dog age: 6–7 years	n=6	—	Percutaneous transplantation	Allogenic AD-MSCs	10^7^	One dog achieved independent walking post-therapy despite the absence of deep pain perception recovery in all animals. This finding suggests a preference for local motor circuit involvement. Regardless of the mechanism, stimulation of local circuits or axonal regeneration—recovery occurred only in chronically paralyzed dogs after therapy	^107^
8.	Chronic SCI	Animal: Dog age: 6–9 years	*n*=3	—	Intralesional transplantation	Human immature dental pulp stem cells (hIDPSs)	1×10^6^	Significant improvements in limb function were noted using the Olby scale, although these were not supported by MRI scans or clinical assessments, suggesting potential limitations in detecting structural changes despite the apparent functional benefits	^96^
9.	Chronic SCI	Animal: Dog age: 1–10 years	*n*=22	*n*=22 Conventional therapy	Percutaneous transplantation	Allogenic BM-MSCs	First: 1×10^6^ Second dose: 1×10^6^, after 15 days interval	The study revealed enhanced proprioception and jumping reflexes in the stem cells group compared to those in the conventional group, indicating improved nerve regeneration. Although there were no significant differences in certain reactions, 50% of paraplegic dogs that received BM-MSC transplantation showed more than 75% recovery according to the Olby score	^90^
10.	Chronic SCI	Animal: Dog age: 2–7 years	*n*=27	—	Intralesional transplantation	AD-MSCs	1×10^7^	The AD-MSC-treated group showed improved motor function; four dogs showed little improvement, and two dogs did not show any improvement. The mechanisms involved in the potential underachievement of the combined therapy are unclear	^105^
11.	Acute thoracolumbar SCI	Animal: Dog age: diverse ages	*n*=11 Stem therapy after t surgical decompression by hemilaminectomy	*n*=11 Surgical decompression by hemilaminectomy	Intralesional transplantation	Allogeneic canine Ad-MSCs	1×10^7^	Ad-MSCs following decompression surgery enhance motor recovery in dogs with acute disc herniation and paraplegia, resulting in faster improvements than those receiving only stem cells. These findings indicate the potential benefits of Ad-MSC therapy for accelerating locomotor recovery	^104^
12	Chronic SCI	Animal: Dog age: 5–11 years	*n*= 8 Two groups with *n*=4 each (SCED and SCED+EA)	*n*=8 Two groups with *n*=4 each (PBS and electroacupuncture (EA))	Intralesional transplantation	Canine exfoliated deciduous teeth (SCED)	2×10^6^	Neurological improvements were noted in some dogs across the various treatment groups. However, the study could not definitively establish a clear positive effect of stem cells or a combination of both stem cells and electroacupuncture	^95^
13	Chronic SCI	Animal: Dog age:4–6 years	*n*=4	—	Intravenous injections	Allogeneic canine BM-MSC (BM-MSC CM)	1×10^6^ Treatment repeated every week for 1 month	Dogs treated with allogeneic BM-MSCs conditioned medium showed clinical improvement. These included better hind limb movement and improved bladder control, as assessed using the Olby locomotors scales at one, three, and 6 months after treatment. Goniometric measurements revealed a partial increase in joint motion range, and two disabled dogs experienced enhanced bladder function	^102^
14	Chronic SCI	Animal: Dog age: Diverse ages	*n*=6	—	Intralesional transplantation	Allogenic BM-MSCs	1×10^6^ Repeated every 15 days till complete recovery to a maximum of four doses	All animals in this study demonstrated notable improvements in weight-bearing neurological functions. Three out of four paraplegic animals achieved complete weight-bearing and mobility, while the remaining dog, although unable to gain weight, showed a significant increase in the recovery score	^91^
15	Chronic SCI (Paraplegia with Intervertebral disc herniation)	Animal: Dog age: 4	*n*=1 year	—	Percutaneous transplantation	Allogenic BM-MSCs	1×10^6^ Repeated every 15 days till complete recovery to a maximum of four doses	A significant improvement was evident after the initial BM-MSC dose, with gradual enhancements observed over time in postural reactions, conscious proprioception, visual/tactile placement, and wheelbarrowing. After two doses, the animal successfully regained the ability to bear full weight on its pelvic limbs, indicating substantial progress in its recovery	^103^
16	Chronic SCI	Animal: dog age: 3–10 years	*n*=8	*n*=4 (Treated with PBS)	Epidural applications (*n*=4) and intralesional transplantation (*n*=4)	Allogeneic transplantation of amniotic membrane-derived stem cells (AMSCs)	2×10^6^ transplanted on days 0, 15, and 45	After 90 days of stem cell therapy, two dogs in the stem cell group regained nociception and proprioception, allowing them to walk. One dog in the surgery plus stem cell group showed some improvement, whereas the other did not recover. No improvements were observed in the placebo group	^94^
17	Chronic SCI	Animal: Dog age: 1–4 years	*n*=4	—	Intralesional transplantation	Allogeneic canine Ad-MSCs	First dose: v5×10^6^ Second dose: 4×10^6^	After the first Ad-MSC transplantation, all dogs showed improved neurological functions within two weeks, gradually progressing over the next 12 weeks. Furthermore, all dogs were free of lumbosacral pain within 1 week, highlighting positive outcomes in terms of pain relief and restoration of neurological function	^110^

## Challenges in the clinical application of MSCs therapy

Preclinical and clinical investigations have shown that xenograft, autologous, and allogeneic stem cell transplantation are viable, safe, and somewhat successful in restoring motor sensory nerve function in animals after SCI. Autologous MSC transplantation has demonstrated superior results compared to allogeneic BM-MSCs in enhancing functional recovery after SCI, as reported by Jung *et al*.^60^. Typically, stem cells are implanted directly into the injured spinal cord intralesionally, before sealing the hemilaminectomy site. Some studies have also explored the percutaneous and intravenous transplantation routes. According to Sharun *et al*.^103^, percutaneous BM-MSC transplantation combined with conventional medicine shows promise in correcting neurological deficits resulting from intervertebral disc herniation in dogs.

Although MSCs are beneficial for neurological recovery following SCI, their effectiveness in SCI treatment remains controversial^113^. The unclear mechanisms of MSC action and cellular processes hindering neural circuit recovery after SCI are significant gaps in our understanding. The survival and long-term viability of transplanted MSCs at the injured site in challenging environments remain unresolved^114^. The fate of transplanted MSCs is dependent on the surrounding cellular microenvironment or niche at the injury site, which impedes their survival^115^. Additionally, the source and origin of harvested MSCs significantly influence their ability to differentiate into specific cell lineages at the injury site, ultimately affecting their therapeutic effectiveness^116^. BM-MSCs can differentiate into both astrocytes and neurons, making them a promising option for treating SCI in animals^25,117–119^. However, a key limitation is that BM-MSCs often undergo multiple *in vitro* cell passages, which can reduce their flexibility and effectiveness, especially with increasing donor age^120^. In contrast, AD-MSCs, UB-MSCs, and WC-MSCs were relatively easy to harvest and culture *in vitro*. In particular, WC-MSCs exhibit superior growth characteristics, proliferation rates, and trilineage differentiation potential^38^. In a comparative preclinical trial of dogs with SCI, MSCs derived from fat, bone marrow, Wharton jelly, and umbilical cord blood contributed to improved functional recovery^61,68^. Notably, umbilical cord blood-derived stem cells demonstrated enhanced nerve regeneration, neuroprotection, and reduced inflammation compared with similar MSCs. However, further clinical trials are necessary to validate these findings.

The limited effectiveness of stem cells in restoring motor sensory nerve function after SCI can be attributed to several factors, including the absence of a mechanical scaffold for guiding and supporting axonal regeneration, presence of inhibitors derived from myelin or reactive glia, and insufficient neurotrophic stimulation and growth factor-mediated neuroprotection^121,122^.

Nanotechnology, an intervention utilizing polymers and stem cell-based scaffolds, offers a potential solution by gradually delivering MSCs to the injury site^88,97,114,123–125^. This technology can facilitate the controlled release of growth factors either as nanoparticles or scaffolds^25,126^. Researchers have explored combining stem cells with growth factors or biomaterials as delivery vehicles or scaffolds in animal studies of SCI therapy^61,93,98,99^. Polymer scaffolds implanted in surgical spinal cord lesions have successfully bridged tissue defects without compatibility issues with host canine spinal cord tissue^71,93^. NSCs remained viable within these scaffolds for at least 2 weeks after surgery, and NSCs migrated into the host spinal cord, suggesting that the scaffolds acted as reservoirs for stem cells and potentially their secretory components^93^. Similarly, the NeuroRegen scaffold demonstrated neurite outgrowths that facilitated the synthesis of new axons and the remyelination of recently repaired axons^100^. Another study by Deng *et al*.^98^ found that adding MSCs to a collagen/heparin sulfate scaffold (CHSS) improved the recovery of dogs with acute complete spinal cord injuries in terms of mobility, axon regeneration, and responsiveness to electrophysiological stimuli. Therefore, combining MSCs with neurotrophic factor codelivery strategies appears to be an effective therapeutic approach for SCI treatment^127^. Various natural and synthetic biomaterials in the form of scaffolds and gels have been developed to mimic the stem cell microenvironment to enhance stem cell survival, proliferation, and differentiation^126^. An ideal biomaterial should exhibit good biocompatibility, low immunogenicity, and biodegradability. Furthermore, when transplanted, they should possess the optimal mechanical properties for cell adhesion and axon regeneration^128^. However, the application of tissue engineering is currently limited to rat SCI models, necessitating further research before clinical implementation. Tissue engineering strategies are expected to overcome the challenges associated with MSC delivery for SCI treatment.

In future research on SCI and MSC-based therapy, it is crucial to address the key aspects of the development of effective clinical practices. The following issues should be studied to define an optimal cell-based therapeutic strategy using MSCs for SCI treatment in both humans and animals.

- Source, administration route, and time window: it is necessary to identify the optimal source of MSCs for SCI treatment and to explore the most effective route of administration as well as the optimal time window for MSC transplantation.
- Dose standardization for lesion size: standardize the dose rate of MSCs for both intralesional and intravenous infusions based on the size of the SCI lesion.
- Optimization of paracrine/neurotrophic factors: optimize the dose and combination of paracrine and neurotrophic factors for cotransplantation with MSCs to enhance therapeutic outcomes.
- Standardization of biomaterials: standardize the use of biomaterials for the delivery, engraftment, or sustained release of paracrine or neurotrophic factors with MSCs at the SCI lesion site. Ensure that biomaterials exhibit properties such as good biocompatibility, low immunogenicity, and suitable mechanical characteristics for cell adhesion and tissue regeneration.
- Post-transplantation therapeutic management: develop a standard procedure for post-transplantation therapeutic management of SCI, including protocols for monitoring, support, and rehabilitation.

## Conclusion

Mesenchymal stem cell therapy holds significant promise for treating SCI, aiming to restore motor and sensory function. However, the efficacy of MSCs in preclinical studies and clinical trials varies, possibly due to factors such as the neuropathology of SCI and the source and dosage of MSCs. Transplanted MSCs play a crucial role in SCI treatment. They not only provide neurons and glial cells but also create an optimal environment for neuroregeneration and angiogenesis at the injury site. MSCs achieve immunosuppression by interacting with immune cells or releasing signaling molecules, thereby reducing inflammation. In addition, they release neurotrophic factors that support axonal regeneration, regulate pathways that inhibit glial scarring, and enhance angiogenesis. Although preclinical and clinical studies have demonstrated improvements in sensory and motor scores in patients, the overall effectiveness of MSC therapy is not sufficient for widespread clinical use. The challenging, toxic environment associated with SCI poses a threat to the survival of the transplanted MSCs. Moreover, the required cell dose and frequency of doses, the mechanism of action of MSCs, and the cellular processes that prevent neural circuit recovery after SCI remain unclear. Therefore, future research should focus on identifying the most effective source of MSCs, determining the optimal cell number and infusion methods, and exploring the potential benefits of cotransplantation with growth factors and biomaterials as delivery vehicles and scaffolds. Such endeavors are crucial for developing effective cell-based therapeutic strategies using MSCs, benefiting both humans and companion animals with SCI.

## Ethical approval

Not applicable.

## Sources of funding

This study received no specific grant from any funding agency in the public, commercial, or not-for-profit sectors.

## Author contribution

B.M.: conceptualization, writing – original draft, writing – review and editing; O.P.C.: conceptualization, data curation, supervision, writing – original draft, and writing – review and editing.

## Conflicts of interest disclosure

The authors declare that there are no conflicts of interests.

## Research registration unique identifying number (UIN)

1. Name of the registry: not applicable for correspondence article.
2. Unique identifying number or registration ID: not applicable.
3. Hyperlink to your specific registration (must be publicly accessible and will be checked): not applicable.

## Guarantor

Om Prakash Choudhary, Associate Professor and Incharge, Department of Veterinary Anatomy, College of Veterinary Science, Guru Angad Dev Veterinary and Animal Sciences University (GADVASU), Rampura Phul, Bathinda 151103, Punjab, India. Tel: +91-9928099090; E-mail: dr.om.choudhary@gmail.com; om.choudhary@gadvasu.in.

## Data availability statement

The data in this research article is not sensitive in nature and is accessible in the public domain. The data is therefore available and not of a confidential nature.
